# Effects of health at every size based interventions on health-related outcomes and body mass, in a short and a long term

**DOI:** 10.3389/fnut.2024.1482854

**Published:** 2024-10-08

**Authors:** Rosario Suárez, Gabriela Cucalon, Carolina Herrera, Martha Montalvan, Jestin Quiroz, Melissa Moreno, Yoredy Sarmiento-Andrade, Luis Cabañas-Alite

**Affiliations:** ^1^School of Medicine, Universidad Técnica Particular del Loja, Loja, Ecuador; ^2^Facultad Ciencias de la Vida, ESPOL Polytechnic University, ESPOL, Campus Gustavo Galindo, Guayaquil, Ecuador; ^3^Universidad Espíritu Santo, Escuela de Medicina, Samborondón, Ecuador; ^4^Faculty of Health Sciences, Miguel de Cervantes European University, Valladolid, Spain

**Keywords:** HAES, nutritional intervention, weight-neutral approach, eating behavior, lifestyle intervention

## Abstract

**Objective:**

This study aims to provide rapid and up-to-date evidence on the effectiveness of Health at Every Size (HAES) interventions compared to controls or other conventional approaches in individuals with overweight or obesity, with the goal of developing more effective and body-diverse respectful strategies.

**Methods:**

A review of literature was carried out using the following databases: PubMed, Scopus, Embase, Web of Science, and SciELO. Research articles were selected based on predefined inclusion and exclusion criteria. Extracted data included study characteristics (design, setting, population demographics, sample size, intervention characteristics, study duration, and follow-up period) and health-related outcomes.

**Results:**

The search yielded 324 articles, of which 20 articles met the inclusion and exclusion criteria. The majority of studies focused on lifestyle improvement, particularly in nutrition, body image, and relationships with food, utilizing a HAES approach. Additionally, other studies examined outcomes such as general well-being, body weight, body composition, cardiovascular risk, and changes in physical activity. Long-term results were particularly noted in studies incorporating physical activity interventions.

**Conclusion:**

HAES interventions appear to be a feasible strategy for promoting overall health and wellness, regardless of body size or shape. However, further evaluation is needed to assess the sustainability of these changes and their long-term impact, as current evidence suggest a they may not be maintained over time.

## 1 Introduction

In both clinical medicine and public health, effective weight management is essential for improving overall health and preventing chronic illnesses (1). Traditional strategies for addressing obesity typically involve weight -reduction methods, such as calorie restriction and increased physical activity (2), which can be implemented through individual or group interventions (3). These methods can offer various health benefits. For example, one study demonstrated that a low-calorie DASH diet can reduce cardiovascular risk factors, as well as lower levels of trimethylamine N-oxide (TMAO), endotoxemia, and chronic inflammation (4). Despite increased attention on obesity treatments, its incidence continues to rise. Several traditional approaches, including diet, exercise, pharmacology, and surgery, have been studied extensively in efforts to address obesity (5).

However, lifestyle change efforts and obesity treatment programs have faced significant challenges, often yielding limited success in sustaining long-term results (4, 6). For instance, while lifestyle interventions have been effective for up to 2 years in individuals with a body mass index (BMI) of under 35 kg/m^2^, many participants experience weight regain, often returning to their pre-intervention weight within 3–5 years (4). Studies estimate that at least 50% of individuals with obesity will regain in the absence of sustained lifestyle changes. One factor influencing this trend is the brain-gut axis, which affects weight through the secretion of gastrointestinal hormones (7).

Furthermore, several organizations recommend intensive treatments, such as bariatric surgery, over drug therapy or lifestyle modifications for individuals with a BMI exceeding 40 kg/m^2^ or between 35 and 40 kg/m^2^ if obesity-related complications are present. However, uncertainties remain regarding the long-term efficacy of surgical procedures (4).

Given the challenges managing obesity, there has been a growing shift in perspective among professionals advocating for a departure from a weight-centric focus. This change has resulted in the rise of Health at Every Size (HAES) movement (8). HAES promotes a nontraditional approach to health and well-being by encouraging individuals to move away from dieting and focus instead on honoring hunger and fullness cues, following a varied, unrestricted diet, and engaging in joyful movement for health promotion—without prioritizing weight loss (9). HAES, which emerged in the early 2000s, also emphasizes mindful and/or intuitive eating, with a focus on body acceptance and overall health rather than weight loss. Key concepts associated with HAES include size acceptance, a non-diet approach, and health-focused behavior changes. The primary goal of HAES is also to reduce weight-based bias and the feelings of guilt often associated with eating and body image. Instead of emphasizing weight loss, HAES focuses on the overall health benefits of behavior changes related to eating and physical activity, highlighting size acceptance and non-dieting.

Studies have shown that implementing the HAES approach can lead to improvements in eating behaviors, such as a decrease in emotional eating, stronger reliance on internal hunger cues, intuitive eating, and improved body satisfaction (9).

Intuitive eating, which is aligned with HAES philosophy, involves responding to internal hunger and satiety cues rather than external signals (10, 11). Studies have found that body acceptance plays a crucial role in this process, with women who perceive body acceptance from others reporting higher self-esteem and better body image. Notably, BMI does not predict a positive body image, but the acceptance of one’s body by significant others and society does (12). Furthermore, greater body acceptance has been linked to enhanced interoceptive awareness, which, in turn, predicts improved body appreciation and success in practicing intuitive eating (12, 13). This approach is particularly important in reducing the risk of eating disorders among adolescents with obesity or weight gain (14). Therefore, this approach can help improve quality of life in the long-term.

Systematic reviews and meta-analyses have reported that HAES interventions can lead to improvements in both total and LDL cholesterol levels in cases of obesity-related malnutrition, as well as psychological benefits like lower depression, enhanced satiety, and a reduction in disordered eating behaviors (15). This review aims to evaluate the effects of HAES intervention on body composition and compare these outcomes with conventional obesity treatments.

Other preventive approaches include the “OPERA Project,” which integrates medical, athletic, gastronomic, and psychological fields to create health promotion interventions in countries with high rates of obesity and overweight (16).

On the other hand, intuitive eating has gained increasing recognition as an alternative to traditional weight-focused interventions. This approach emphasizes responding to internal cues of hunger and satiety, encouraging individuals to develop a more positive and mindful relationship with food (17). Recent studies have shown a strong association between intuitive eating and improved psychological outcomes, such as enhanced body image and reduced psychological distress, particularly in populations at risk of developing eating disorders (18) One study demonstrated that women practicing intuitive eating experienced better body image and reduced psychological distress, further supporting its potential as a viable intervention for promoting mental health and overall well-being, regardless of obesity history (19).

Research also emphasizes the interconnectedness between body acceptance, self-esteem, and intuitive eating. Higher levels of body acceptance predict greater success in intuitive eating, creating a positive feedback loop between body positivity and healthy eating behaviors (20). Moreover, individuals who perceive greater acceptance of their bodies—both from themselves and from others—tend to exhibit improved interoceptive awareness, enhanced body appreciation, and a higher likelihood of engaging in intuitive eating practices (21). These results highlight the significance of focusing on body image and self-perception in interventions aimed at promoting intuitive eating.

This review aimed to compare the effects of HAES-based interventions with those of conventional approaches in individuals with overweight or obesity.

## 2 Methodology

### 2.1 Literature research

This review was carried out according to the PRISMA 2020 statement guidelines (17). An comprehensive search was performed in PubMed, Scopus, Embase, Web of Science, and SciELO, identifying 324 articles published between January and May 2024. The search strategy was developed using the PICOS method, incorporating key terms such as “Health at Every Size” (HAES), “intuitive eating,” “non-weight-centrism,” “overweight,” “obesity,” and “fat mass.” The search syntax and controlled vocabulary for each database were adjusted accordingly, and the complete search strategy is provided in the Supplementary Table S1. This approach enabled a broad exploration of studies relevant to health and nutrition in populations with overweight and obesity.

### 2.2 Study eligibility, selection, and data extraction

For inclusion in the review, research articles were selected based on the following criteria: (1) BMI or Waist-Hip Index (WHI) indicative of obesity, (2) study participants over the age of 18, (3) articles written in English, Spanish, or German, (4) original research interventions primarily aimed at improving and reporting on the effects of body composition, health, or behavioral outcomes of individuals, using interventions based on the HAES (Health at Every Size) method. Eligible studies included randomized controlled trials (RCTs), quasi-experimental interventions, pre-post studies, and feasibility trials, and (5) with a publication date between 2013 and 2023.

Exclusion criteria were as follows: (1) studies that did not address the primary objective of the review, (2) articles focusing solely on hospitalized patients, (3) duplicate studies already included in another database, (4) studies using pharmacological or surgical interventions, (5) systematic reviews, narratives, meta-analyses, and protocols, and (6) interventions focused on specific diseases or health behaviors not related to body weight.

From an initial pool of 324 articles, 131 duplicates were identified and removed, along with five entries that were books and did not meet the inclusion criteria for original research. This left 188 articles for further screening. Following an initial screening of titles and abstracts, 97 articles were excluded due to study type (e.g., reviews, editorials, protocols) or lack of originality. This left 91 references for further detailed screening. During this phase, 69 articles were excluded for failing to meet the inclusion criteria: five due to involving participants under 18 years old, eight due to focusing on non-obese populations, 32 because of unsuitable study designs, and 26 because their objectives differed from the review’s scope.

The final selection of articles was carried out through a comprehensive review process conducted by three independent reviewers. All selection discrepancies were addressed through discussion and agreement. Throughout the review, Zotero software was used for reference management. Data extraction was conducted by two reviewers and verified by all authors to ensure consistency and accuracy. The data extracted included study design, population characteristics, intervention details, and health outcomes.

## 3 Results

**Figure 1:**
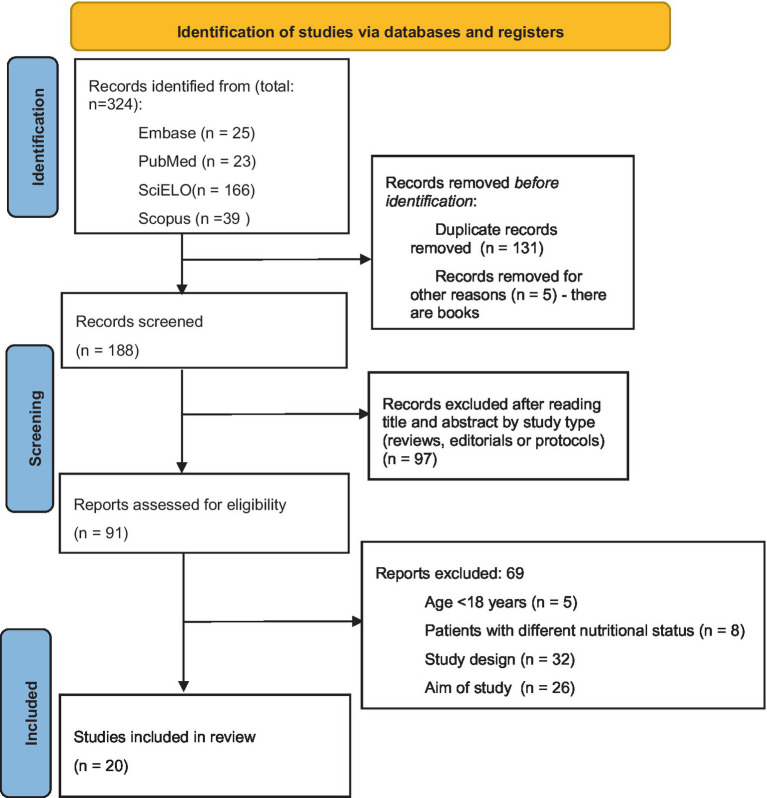
Study data collection: authors’ own creation.

Twenty articles that satisfied the inclusion criteria were selected for this report and are shown in Table 1. The screening process is illustrated in Figure 1, which provides a flow diagram of the search strategy.

**Table 1:** Results summary.

Autor (year)	*n*	Design	Objective	Intervention	Time	Results	Conclusions
Carbonneu et al. (2017) (22)	Intervention: 216 women, Observational: 110 women	Longitudinal	Investigate the effects of a HAES^®^ program on intuitive eating and diet quality in women.	Program with an emphasis on body acceptance and intuitive eating: thirteen 3 h weekly meetings and a 6 h intensive day in groups	16 months	HAES^®^ program ↑ score at *T* = 4 months in quality. The daily consumption of high-fat/ high-sugar foods did not differ between the two groups	Improved the quality of their food intakes at short but not long term, and that their diet quality was positively related to intuitive eating score.
M. Punna et al. (2021) (27)	*N* = 177	Longitudinal	Discover whether an ACT (acceptance and commitment therapy)-based peer-tutored online intervention can increase self-reported physical activity.	Program provided by health services, including three online modules of ACT of six week each, and via five group meetings and four phone calls.	24 months.	Baseline: High profile group: ↑ physical activity, psychological flexibility and ↓ thought suppression, psychological symptoms measured by DASS. During the intervention: Low profile group: ↑ Physical activity High profile group: ↑ Psychological flexibility (AAQ-II) ↓ thought suppression (WBSI) in both profiles	Intervention was effective for participants with low physical activity participation.
Dimitrov Ulian, M et al. (2022) (33)	*N*: 55	Randomized controlled trial.	Investigate the association between weight loss resulting from Health at Every Size (HAES^®^)-based interventions and changes in cardiometabolic risk factors.	HAES^®^-based interventions	7 months	Weight loss was associated ↓ in waist circumference, fasting glycemia, total cholesterol LDL cardiometabolic risk, ↑ quality of life	Improvements in cardiovascular risk factors and quality of life regarding the change of weight.
Carroll et al. (2007) (20)	*N* = 31	Secondary analysis of a randomized, controlled trial.	Examined the short-term effects of a non-dieting lifestyle intervention program, within the theoretical psychological framework of self-determination theory (SDT)	Non-dieting lifestyle intervention	3 months.	↑ Metabolic improvements: diastolic blood pressure and high-density lipoprotein cholesterol in both groups. The lifestyle intervention group: ↑ general psychological well-being	Improvements of psychological well-being improved cardiorespiratory fitness and psychological well-being.
Borkoles et al. (2016) (4)	*N* = 31	RCT	Examine the effects of a non-dieting lifestyle intervention designed in the frameworks of Health at Every Size and self-determination theory on weight maintenance and psychological well-being.	2 h orientation session on weight management, healthy eating, and physical activity	12 months.	↓ weight at 3 months for IG. SPP showed significant interaction effects; **12 months** Significantly improved ↑ psychological functioning in autonomy ↓ Chance Subscale from baseline to 12-month follow-up Perceived stress ↓ 12 months	The role of a non-dieting weight management approach by including several important psychological dimensions such as general well-being and a multidimensional measure of self-esteem.
Mensinger et al. 2023 (28)	*N* = 40	Longitudinal.	Effectiveness of a weight-inclusive health intervention aimed at reducing disordered eating through the promotion of intuitive eating	Face-to-face group sessions in the weight-inclusive health program.	6 months.	The total effects of the weight inclusive health program ↓ uncontrolled eating, and ↓ emotional eating	Importance of minimizing the self-shame and blame that is inherent in internalized weight stigma and fuels maladaptive eating behavior.
Scagliusi F et al. (2020) (31)	*N* = 39	RCT.	Describe qualitatively the responses to weight stigma and body acceptance issues from urban Brazilian gorda women.	HAES^®^ program.	24 months.	The I-HAES^®^-group: ↑ body acceptance, well-being, CTRL-group internalized and accepted stigma.	HAES^®^ could meaningfully address weight stigma and promote body acceptance
Dimitrov Ulian M et al. (2018) (9)	*N* = 58	RCT	Investigate the effects of an intensive, interdisciplinary HAES^®^ -based intervention on multiple physiological aspects.	HAES^®^ program	7 months.	HAES^®^ group: ↑ maximal oxygen uptake and better performance on the timed stop test ↑ dietary attitudes and practices. ↑ Body Attitude improvement ↑ physical health ↑ psychological health ↑ quality of life	HAES^®^ improved participants dietary attitudes and practices, body image perception, physical capacity, and health-related quality of life, despite a lack of change in body weight and physical activity levels.
Gagnon-Girouard MP (2010) (21)	*N* = 107	RCT	To compare the effects of a HAES intervention with and a no-intervention control group	HAES intervention (*N* = 48), (2) social support (SS) group intervention (*N* = 48), and (3) waitlist (WL) (*N* = 48).	12 months.	↑ psychological improvement	HAES improved with psychological variables and body weight maintenance
Mensinger et al. (2016) (1)	*N* = 80:	RCT	Compared the effectiveness of a weight-neutral versus a weight-loss program for health promotion.	HUGS Program for Better Health LEARN Program for Weight Management	24 months	↓ Weight loss, BMI and LDL cholesterol levels = Blood pressure Fasting blood glucose, and triglyceride	Provides novel evidence supporting an alternative approach to weight loss in the promotion of health for high BMI individuals.
Cloutier-Bergeron (2019) (26)	*N* = 210	Multicentric quasi experimental study.	Identify trajectories of responses to a non-diet intervention for adult overweight/obese women.	13 weekly sessions of 3 h plus an intensive 6 h day led by a social worker or psychologist and a dietitian.	16 months.	Non-responders: ↑ weight gain in the three months (*p* = 0.03); ↑ depressive symptoms, ↓ quality of life and self-esteem.	There is a need to consider psychological characteristics to move towards personalized healthcare in obesity management.
Provencher et al. (2009) (18)	*N* = 144;	RCT	To assess the effects of HAES intervention on eating behaviors, appetite sensations, metabolic and anthropometric variables, and physical activity levels in women.	The HAES: 14 weekly sessions. The SS intervention: The control group: usual lifestyle habits for the duration of the study.	12 months.	Situational susceptibility to disinhibition and susceptibility to hunger: ↓ in both groups ↓ in both groups ↓ weight at 16 months HAES	HAES approach could have long-term beneficial effects on eating behaviors.
Bacon et al. (2005) (19)	*N* = 78	RCT	Examine a model that encourages HAES as opposed to weight loss.	HAES program or diet program	24 months	The 92% of the HAES group completed the program. The diet group: ↓ weight posttreatment and maintained the weight loss. ↓ BP posttreatment and post aftercare. The HAES group sustained change at follow-up	HAES could maintain long-term behavior change
Jospe et al. (2017) (23)	*N* = 250	Exploratory secondary analysis from RCT	Examine the adherence to “hunger training” influenced weight loss and eating behavior	Hunger training, which monitoring blood glucose levels before eating to teach individuals to eat only when truly hungry. Participants received diet and exercise counseling in a face-to-face session.	6 months.	Hunger training ↓ weight over a 6-month period.	Hunger training is a feasible and effective method for weight loss and improving eating behaviors in adults, provided that adherence guidelines are met.
Begin et al. (2019) (25)	*N* = 216	Quasi-experimental	To assess the effects of HAES intervention on eating behaviors, psychological factors, and BMI	The Health at Every Size (HAES) intervention = 14 weekly meetings provided by health professionals, the program focused on promoting healthy lifestyle habits, self-acceptance, and intuitive eating.	16 months	HAES group: ↑ intuitive eating scores, ↓ obsessive-compulsive eating scores, ↑ the flexible restraint scores, ↓ disinhibition and susceptibility to hunger scores at 4–16 months.	HAES intervention led to significant improvements in psychological outcomes such as self-esteem, body esteem, and depressive symptoms, as well as positive changes in eating behaviors like intuitive eating, disinhibition, and susceptibility to hunger; and was effective in improving eating-, weight-, and psychological-related variables in the short and long term.
Mensinger et al. (2016) (1)	*N* = 80	RCT	Examine the impact of internalized weight stigma on eating behaviors in women with high BMI	HUGS Program for Better Health and Health at Every Size^®^.	24 months.	Effect of restraint: ↑ weight control program. Internalized weight stigma: ↑ in both groups at 6–24 months	Incorporate more innovative and direct methods to reduce internalized weight stigma for women with high BMI in order to enhance the overall benefits of weight-neutral approaches.
Ulian et al. (2015) (30)	*N* = 30	Prospective	To evaluate the effects of a non-prescriptive multidisciplinary intervention based on the Health at Every Size^®^ philosophy in obese women	Weekly physical activity sessions, five philosophical workshops, and bimonthly individual nutritional sessions.	12 months	↑ attitudes towards eating, body image perception, and physical activity, empowered to make changes, and exercise	Non-prescriptive multidisciplinary intervention based on the Health at Every Size^®^ philosophy was effective in improving various aspects of participants’ well-being, including blood pressure, lipid profile, physical activity levels, eating behaviors, self-esteem, and body image perception, despite no significant weight loss.
Berman M. et al. (2022) (32)	*N* = 19	RCT	Comparing AY to a more commonly used and widely disseminated group-based behavioral weight loss program, WW.	Group-based behavioral weight loss program	12 months.	Fitness ∅ groups ↑ eating disorder symptoms and ↑ in WW.	AY appeared safe, feasible, and offered initial evidence of efficacy for depression.
Sabatini F. et al. (2019) (2)	*N* = 43	RCT	Investigated the perceptions of obese women about eating pleasure before and after an intervention based on the HAES approach.	HAES intervention	7 months.	The HAES group: ↑ autonomy regarding eating, pleasure in shared meals, familiarity with cooking practices, ↓ automatic eating.	The HAES- enhance appreciation for physical activity, and stimulation of pleasure eating without leading to indiscriminate eating.
Mensinger J. and Meadows A. (2017) (29)	*N* = 80	RCT.	To investigate the influence of internalized weight stigma (IWS) on physical activity (PA) outcomes among women	Health-at-every-size vs. weight-loss-focused group-based healthy living program.	6 months	↑ enjoyment of moderate physical activity ↑ reduced internalized weight stigma	Self-directed stigma and holding negative attitudes about one’s weight interferes with positive changes in PA outcomes.

Characteristics of included studies are summarized in Table 1.

Of the final articles included, twelve described programs focused on lifestyle improvements, specifically related to nutrition, body image, or the relationship with food, all framed within a HAES perspective (1, 18–28). Eight studies adopted the same perspective or intervention but included physical activity (2, 4, 9, 29–33). In addition, eight studies investigated overall well-being (1, 2, 4, 9, 20, 21, 31, 33), ten focused on body weight, body composition, and body image (4, 9, 18–21, 29, 33), five examined cardiovascular risk impacts (1, 9, 19, 26, 33), two explored changes in eating behaviors (28, 30) and three analyzed physical activity outcomes (27, 29, 30).

Furthermore, eight studies evaluated the long-term effects of lifestyle changes in participants undergoing these interventions (1, 4, 18, 19, 21, 22, 25, 26).

These findings will be analyzed in detail in the following section, comparing them to similar studies for further insights.

## 4 Discussion

This review aimed to explore the feasibility and effectiveness of HAES interventions for individuals with obesity, focusing on their impact on various health-related outcomes. We reviewed 20 articles; while preliminary findings indicate promising short-term outcomes across various health domains, our analysis focuses on long-term effects.

### 4.1 Effects on overall well-being

Eight studies evaluated the effects of HAES on quality of life or overall well-being using different tools such as validated instruments, focus groups, and interviews. One consistent finding across these studies is that strategies aimed at enhancing overall well-being can positively affect health outcomes, irrespective of significant weight loss.

In the study by Ulian et al. (9), both the intervention group (I-HAES^®^) and the control group (CTRL) showed significant improvements in quality-of-life parameters. The I-HAES^®^ group demonstrated increases in the “physical” (*p* = 0.05), “psychological” (*p* = 0.03), and “quality of life” (*p* = 0.02) domains, while the control group improved in the “psychological health” (*p* = 0.04) and “perception of quality of life” (*p* = 0.01) domains. These results were achieved as participants worked to change their routines.

In a follow-up study conducted in 2022 with the same population, weight loss was associated with improved quality of life (*β* = −1.05, *p* = 0.007), as measured by the World Health Organization Quality of Life—BREF questionnaire (WHOQOL-BREF). This intervention primarily focused on nutritional counseling without a diet prescription (33).

The results suggest that the positive changes in body image observed in the traditional control group were expanded in the interdisciplinary intervention group, which likely fostered an empathetic atmosphere, increased self-esteem, and improvements in body attitudes and perception, all of which contributed to an enhanced quality of life, which might be a result of these improvements. In contrast, Brown et al. (34), highlighted the negative effects of stigma and lack of support in primary care settings on access to healthcare services for patients with obesity, emphasizing the importance of including nutritional counseling in obesity treatment programs.

Scagliusi et al. (31), did not present comparative data due to their study design, which did not account for time effects versus intervention impacts. However, Sabatini et al. using a case–control design and focus groups, found that both intervention groups reported improvements in eating pleasure, a decrease in guilt around eating, and enhanced experiences of commensality. The I-HAES group also reported reductions in emotional eating, greater confidence in food choices, improved cooking skills, and less mindless eating. These studies underscore the need for standardized tools to measure quality-of-life outcomes.

Carroll et al. (20) reported significant improvements in psychological well-being compared to the control group (test for interaction, *p* = 0.0005) in absence of significant changes in body mass or composition. Similarly, Borkoles et al. (4) assessed well-being using the General Well-Being Schedule (GWB) and found significant improvements across all subscales from baseline to 12-month follow-up, despite the absence of significant weight loss. Similarly, The Healthy Weight in Lesbian and Bisexual Women study (*n* = 266 LB women age ≥ 40) conducted by Ingraham (35), assessed the effects of mindfulness interventions on health outcomes. Although weight loss was not a primary outcome, increased mindfulness was associated with significant improvements in mental health and quality of life.

Gagnon-Girouard et al. (36) observed no significant short-term changes in well-being but reported that HAES interventions could lead to sustained long-term improvements in mood, self-esteem, quality of life, body dissatisfaction related to appearance, body dissatisfaction—weight, body dissatisfaction—attribution, binge eating, and body weight. These improvements are directly targeted by the HAES intervention, suggesting that achieving self-acceptance, enhanced quality of life, and positive body image may require a longer duration to manifest.

Finally, Mensinger et al. (24) found sustained improvements in psychological well-being, such as enhanced quality of life and self-esteem, over 24 months, though there were not significant differences between the intervention and control groups. Bruce and Ricciardelli (37) reviewed the growing body of literature supporting a positive correlation between intuitive eating and emotional well-being in women. Similarly, Tribole and Resch, in their book on intuitive eating, they associated restrictive eating behaviors with an increase in depressive symptoms and poor emotional regulation (38).

### 4.2 Effects on body weight, body composition and body image

Several authors have reported the impact of the HAES intervention on body weight after. Ulian et al. (33) noted that after 7 months of intervention, weight loss was significantly related to improvements in waist circumference (*β* = 0.83, *p* < 0.001). Similarly, Borkoles et al. (4) also found a modest weight reduction after 3 months of lifestyle intervention.

Other authors reported that improvements in body esteem during the intervention phase suggested the likelihood of maintaining body weight during follow-up phase (*p* = 0.011) (21). Mensinger et al. (1) observed reductions in weight, BMI, and LDL cholesterol from baseline to post-intervention (*p* = 0.003), with greater reductions in the weight-neutral program. Provencher et al. (18) found that 63.4% of women in the HAES group maintained a lower weight at 16 months compared to baseline (mean BMI 30.10.4 at baseline vs. 29.50.5 at 16 months; 2% difference from the initial weight).

In contrast, Bacon et al. (39) reported significant weight loss in the diet group post-treatment (−5.2 kg ± 7.3 from baseline), with participants maintaining a 5.2% reduction in weight after follow-up (−5.3 kg ± 6.7 from baseline). However, most studies noted weight maintenance or even weight gain over time (1, 4, 19, 33). Additionally, some authors using HAES methods reported no significant changes in body weight (9, 20, 23, 26, 29).

Despite these findings, several reviews highlight that HAES and other non-weight-centric interventions lead to significant and sustained changes in dietary behaviors and practices (up to 2 years), although these changes are not reflected in anthropometric measurements (9, 40). These results suggest that weight is not the sole indicator of overall well-being.

### 4.3 Impact on cardiovascular risk

Analyzing the results of the aforementioned studies reveals a variety of findings concerning the HAES approach and its impact on cardiovascular health. Ulian et al. (33) found that weight loss was associated with significant improvements in waist circumference (B = 0.83; *p* < 0.001), fasting blood glucose (B = 0.45; *p* = 0.036), total cholesterol (B = 1.48; *p* = 0.024), LDL (B = 1.33; *p* = 0.012), and pooled cardiometabolic risk (B = 0.18; *p* = 0.006). In contrast, Mensinger et al. (1) observed no significant changes in systolic or diastolic blood pressure, fasting blood glucose, or triglyceride levels over time. Cloutier-Bergeron et al. (26) identified significant differences when comparing non-responders to other groups, finding higher rates of cardiovascular disease (x2 (1, *N* = 205) = 6.232, *p* = 0.013, n2 = 0.03) and dyslipidemia (x2 (1, *N* = 199) = 5.471, *p* = 0.019, n2 = 0.028). Bacon et al. (19) reported a significant decrease in systolic blood pressure but no significant changes in diastolic blood pressure. In addition, Ulian et al. (9) showed that although fat-free mass (FFM) increased and waist and hip circumferences slightly decreased, these changes were not statistically significant.

Recent studies support the complexity of the relationship between the HAES approach and cardiovascular risk. For example, Schaefer and Magnuson (41) revealed significant improvements in health-related quality of life (B = 0.72; *p* = 0.021) and a reduction in anxiety (B = −1.25; *p* = 0.038) in individuals who adopted a HAES approach. Similarly, Bacon and Aphramor (42) found a significant decrease in perceived stress (B = −0.56; *p* = 0.017) and improved body image in participants following a health-focused approach rather than a weight-loss-centered one.

Gagnon-Girouard et al. (21) reported that the HAES approach improved self-esteem (B = 0.72; *p* = 0.021) and reduced depressive symptoms (B = −1.25; *p* = 0.038) in women with overweight or obesity. Mensinger et al. (1) also found significant improvements in physical activity (B = 0.56; *p* = 0.017) and life satisfaction in participants following the HAES approach.

These results emphasize the importance of considering comprehensive approaches, such as HAES, to promote cardiovascular health and overall well-being. They highlight the need to personalize health strategies to address cardiovascular risk effectively in diverse populations, focusing on improving health and quality of life rather than just weight loss.

### 4.4 Impact on eating behaviors

Mensinger et al. (28), provide significant statistical supporting the importance of addressing maladaptive eating patterns in women with high BMI. Their study demonstrated a 25% decrease in emotional eating scores (b = −1.79, SE = 0.34, *p* < 0.0001), and a 20% reduction in uncontrolled eating behaviors (b = −3.76, SE = 0.64, *p* < 0.0001), following 6 months of participation in a HAES-based program, showing a statistically significant improvement in dietary patterns (*p* < 0.05) (28).

Moreover, each reduction in weight stigma was associated with a corresponding 0.75-point increase in intuitive eating behavior score (*r* = −0.60, *p* < 0.01), highlighting that reducing weight stigma is crucial for fostering a healthier relationship with food, an essential aspect of the HAES intervention.

Ulian et al. (30) also demonstrated the impact of the HAES approach by showing a 13% decrease in body fat mass, an 11.1% reduction in body fat percentage, a 3.6 kg reduction in weight, and a 3.2 -point reduction in BMI, all of which were statistically significant. Additionally, binge eating behaviors improved; initially, 57.1% of participants exhibited moderate binge eating, but by the end of the study, 78.6% exhibited no binge eating behaviors, while only 14.3% continued to display moderate levels of such behaviors.

Both studies highlight the importance of comprehensive approaches that address both physical and psychological aspects of health during interventions. Mensinger et al. (28) provide evidence of reduced uncontrolled and emotional eating, while Ulian et al. (30) show reductions in binge eating. These findings suggest that reducing weight stigma and promoting intuitive eating can lead to improved health outcomes and greater body acceptance.

### 4.5 Effects on physical activity

Punna et al. (27) investigated the effects of an online peer-mentored intervention based on acceptance and commitment therapy. The study found that participants with low baseline physical activity levels significantly increased their participation in physical activity, highlighting the importance of personalizing interventions to maximize effectiveness.

Ulian et al. (9) also reported significant improvements in body composition following an exercise program, nutritional counseling, and philosophical workshops. After the intervention, there were significant reductions in weight, BMI, body fat mass, and fat mass percentage (−3.6, −3.2%, −13.0%, and −11.1%, respectively; *p* ≤ 0.05), all of which positively impacted cardiovascular and metabolic health. Similar findings were reported by Schaefer and Magnuson (41), who found that participants adopting a HAES approach, including physical activity, experienced significant improvements in health-related quality of life (B = 0.72, *p* = 0.021) and reduced anxiety (B = −1.25, *p* = 0.038). Bacon and Aphramor (42) similarly found that combining physical activity with nutrition education led to significant reductions in perceived stress (B = −0.56; *p* = 0.017) and improvements in body image. Gagnon-Girouard et al. (21) also showed that health promotion strategies incorporating physical activity increased self-esteem (B = 0.72; *p* = 0.021) and reduced depressive symptoms (B = −1.25; *p* = 0.038) in women with overweight or obesity.

These results highlight the importance of adopting a holistic approach that integrates physical activity with other wellness factors to foster positive changes in health and quality of life. More recently, Mensinger et al. (1) demonstrated that a “weight neutral” approach, which emphasizes physical activity and overall health, was associated with greater body satisfaction (B = 0.72; *p* = 0.021) and a reduction in eating disorder symptoms (B = −1.25; *p* = 0.038) in women with overweight or obesity. These findings further support the value of prioritizing health and wellness over weight loss.

Schaefer and Magnuson (41) supported these findings, reporting significant improvements in self-efficacy for exercise (*β* = 0.72; *p* = 0.021) and increased physical activity adherence (β = −1.25; *p* = 0.038). These results underscore the importance of fostering self-efficacy and intrinsic motivation to promote the adoption and long-term maintenance of an active lifestyle.

In contrast, Mensinger and Meadows (29) observed that the effect of internalized weight stigma on moderate-intensity physical activity was not statistically significant (b = 0.22, SE = 0.11, 95% CI, *t*(132) = 1.89, *p* = 0.061). However, they noted a significant increase in moderate-intensity physical activity over time (b = 0.80, SE = 0.14, 95% CI [0.51, 1.08], *t*(71) = 5.54, *p* < 0.001), emphasizing the importance of considering psychological factors such as weight stigma to promote long-term physical activity adherence.

Bacon et al. (19) further revealed that the HAES approach, which combines physical activity and nutrition education, significantly improved diet quality (B = 0.72; *p* = 0.021) and reduced cardiometabolic risk (B = −1.25; *p* = 0.038) in adults with overweight or obesity. These findings highlight the value of adopting a holistic approach to promote cardiovascular and metabolic health.

### 4.6 Results at long term

A very relevant question regarding health interventions, especially those addressing cardiovascular, metabolic, or nutritional health, is whether the results are maintained in the long term. In other words, do individuals continue adhering to the changes implemented during the intervention? This question has been extensively explored in the articles included in this review, and the findings are discussed below.

The average duration of the interventions analyzed ranges from 4 to 6 months, while long-term analysis typically take place 12 months after the intervention’s completion (4, 18, 22, 25, 26, 36), or in some cases, up to 24 months (1, 19).

Some interventions showed that changes in eating behaviors, dietary quality, or weight were not maintained over the long term (18, 24), or that the differences between control and intervention groups were no longer significant after the intervention period (1, 4, 18). However, results in areas like autonomy and other psychological functions remain significant (*p* < 0.001) (4), and improvements in depression symptoms were observed (*p* < 0.05) (25, 26). Additionally, changes in individual’s relationships with food, such as reduced compulsive eating (*p* < 0.0001), were sustained (25). In contrast, studies focusing on quality-of-life improvements found that these effects were not maintained long-term (30). Cloutier-Bergeron et al. (26) also highlighted the existence of a group classified as “non-responders,” who exhibited worse psychological function, less adaptation to changes in eating behavior, and higher rates of clinical depression. These individuals struggled to adjust to the behavioral changes introduced during the intervention.

While interventions based on this methodology can have a significant effect in the short-term impact, they seem to be less effective in the long term. Research evaluating HAES interventions, which emphasize healthy behaviors, size acceptance, and non-dieting approaches, has demonstrated health benefits regardless of weight loss. Nevertheless, as mentioned in previous sections and corroborated by other literature from the last twenty years (33, 43, 44), the long-term effectiveness of these interventions remains in question. Available data and findings from other authors suggest the long-term (>12 months) may not be sustained (45, 46). More long-term data are needed to support the use of these interventions.

It is important to acknowledge both the strengths and limitations of this review. One limitation is that some studies were carried out by the same research teams, meaning more studies from various regions are needed to strengthen the evidence. While interventions have been carried out in Canada, Brazil, the UK and the USA, which provides some diversity, more information is needed on the application of HAES across different cultural contexts. The increasing number of participants in these studies makes the data more robust, but it should also be noted that the majority of these studies have focused exclusively on women. This gender bias indicates that more research is needed on the effects of these interventions on men. Furthermore, few studies evaluate body composition or weight measurements, aligning with HAES principles of weight-neutrality. Nonetheless, this approach complicates comparisons between clinical outcomes.

Future research should include men and older populations, have longer follow-ups (>12 months), extend the intervention duration (>6 months), and involve larger population groups. Additionally, more detailed data on food intake is needed to understand what nutritional changes lead to improved health outcomes and, where applicable, reduced weight or body fatness in intervention groups. Factors such as energy-balance, food intake, and overall well-being, should be more thoroughly examined.

In conclusion, available data suggest that HAES interventions have positive effects on body composition, cardiovascular health, and psychological factors related to food and well-being. However, these long-term effects (particularly in studies lasting a minimum of 2 years) require further verification, as current evidence suggests that some benefits may not be sustained. More extensive and longer-term studies are needed to clarify these findings.
