# Supplementary material for: Effects of health at every size based interventions on health-related outcomes and body mass, in a short and a long term
Source: Front Nutr. 2024 Oct 8;11:1482854. doi: 10.3389/fnut.2024.1482854 (PMC11493725; doi:10.3389/fnut.2024.1482854)
Supplement: Supplementary file 1 [file Table_1.docx]

***Supplementary Material***

# Table. Search Strategy

The bibliographic routes used for the scientific literature search engines indicated in section "2.1 Literature Research" of the work presented are indicated below.

| PubMed | (("Health at every size") OR ("Health-at-every-size") OR ("HAES")) AND (("weight-neutral" OR "weight-inclusive" OR "weight acceptance" OR "size acceptance" OR "body acceptance" OR "non-diet" OR "anti-diet")) AND (("overnutrition*") OR ("Obesity"[Mesh] OR "Obes*" OR "Obesity, Abdominal"[Mesh] OR "Abdominal obesity" OR "Obesity, Morbid"[Mesh] OR "Morbid Obesity") OR ("adipos*" OR "anthropometr*" OR "Waist hip ratio" OR "skinfold thickness")) AND ("Epidemiologic Studies"[Mesh] OR "Epidemiologic Studies" OR "Epidemiologic Study" OR "Cohort Studies"[Mesh] OR "Cohort Studies" OR "Cohort Study" OR “Historical Cohort Studies” OR “Historical Cohort Study” OR “Cohort Analyses” OR “Cohort Analysis” OR “Closed Cohort Studies” OR “Closed Cohort Study” OR “Incidence Studies” OR “Incidence Study” OR "Longitudinal Studies"[Mesh] OR "Longitudinal Studies" OR "Longitudinal Study" OR “Longitudinal Survey” OR “Longitudinal Surveys” OR "Follow-Up Studies"[Mesh] OR "Follow-Up Studies" OR "Follow-Up Study" OR “Followup Studies” OR “Followup Study” OR "Prospective Studies"[Mesh] OR "Prospective Studies" OR "Prospective Study" OR "Case-Control Studies"[Mesh] OR “Case-Control Studies” OR “Case-Control Study” OR “Case-Comparison Studies” OR “Case-Comparison Study” OR “Case-Compeer Studies” OR “Case-Compeer Study” OR “Matched Case-Control Studies” OR “Matched Case-Control Study” OR “Nested Case-Control Studies” OR “Nested Case-Control Study”) |
| --- | --- |
| Scopus | (("Health at every size" ) OR (Health-at-every-size ) OR (HAES )) AND ((weight-neutral OR weight-inclusive OR "weight acceptance" OR "size acceptance" OR "body acceptance" OR non-diet OR anti-diet )) AND ((overnutrition* ) OR (INDEXTERMS(Obesity) OR Obes* OR INDEXTERMS("Obesity, Abdominal") OR "Abdominal obesity" OR INDEXTERMS("Obesity, Morbid") OR "Morbid Obesity" ) OR (adipos* OR anthropometr* OR "Waist hip ratio" OR "skinfold thickness" )) AND (INDEXTERMS("Epidemiologic Studies") OR "Epidemiologic Studies" OR "Epidemiologic Study" OR INDEXTERMS("Cohort Studies") OR "Cohort Studies" OR "Cohort Study" OR "Historical Cohort Studies" OR "Historical Cohort Study" OR "Cohort Analyses" OR "Cohort Analysis" OR "Closed Cohort Studies" OR "Closed Cohort Study" OR "Incidence Studies" OR "Incidence Study" OR INDEXTERMS("Longitudinal Studies") OR "Longitudinal Studies" OR "Longitudinal Study" OR "Longitudinal Survey" OR "Longitudinal Surveys" OR INDEXTERMS("Follow-Up Studies") OR "Follow-Up Studies" OR "Follow-Up Study" OR "Followup Studies" OR "Followup Study" OR INDEXTERMS("Prospective Studies") OR "Prospective Studies" OR "Prospective Study" OR INDEXTERMS("Case-Control Studies") OR "Case-Control Studies" OR "Case-Control Study" OR "Case-Comparison Studies" OR "Case-Comparison Study" OR "Case-Compeer Studies" OR "Case-Compeer Study" OR "Matched Case-Control Studies" OR "Matched Case-Control Study" OR "Nested Case-Control Studies" OR "Nested Case-Control Study" ) |
| Embase | (('Health at every size' ) OR (Health-at-every-size ) OR (HAES )) AND ((weight-neutral OR weight-inclusive OR 'weight acceptance' OR 'size acceptance' OR 'body acceptance' OR non-diet OR anti-diet )) AND ((overnutrition* ) OR (Obesity/exp OR Obes* OR 'Obesity, Abdominal'/exp OR 'Abdominal obesity' OR 'Obesity, Morbid'/exp OR 'Morbid Obesity' ) OR (adipos* OR anthropometr* OR 'Waist hip ratio' OR 'skinfold thickness' )) AND ('Epidemiologic Studies'/exp OR 'Epidemiologic Studies' OR 'Epidemiologic Study' OR 'Cohort Studies'/exp OR 'Cohort Studies' OR 'Cohort Study' OR 'Historical Cohort Studies' OR 'Historical Cohort Study' OR 'Cohort Analyses' OR 'Cohort Analysis' OR 'Closed Cohort Studies' OR 'Closed Cohort Study' OR 'Incidence Studies' OR 'Incidence Study' OR 'Longitudinal Studies'/exp OR 'Longitudinal Studies' OR 'Longitudinal Study' OR 'Longitudinal Survey' OR 'Longitudinal Surveys' OR 'Follow-Up Studies'/exp OR 'Follow-Up Studies' OR 'Follow-Up Study' OR 'Followup Studies' OR 'Followup Study' OR 'Prospective Studies'/exp OR 'Prospective Studies' OR 'Prospective Study' OR 'Case-Control Studies'/exp OR 'Case-Control Studies' OR 'Case-Control Study' OR 'Case-Comparison Studies' OR 'Case-Comparison Study' OR 'Case-Compeer Studies' OR 'Case-Compeer Study' OR 'Matched Case-Control Studies' OR 'Matched Case-Control Study' OR 'Nested Case-Control Studies' OR 'Nested Case-Control Study' ) |
| Web of Science | (("Health at every size" ) OR (Health-at-every-size ) OR (HAES )) AND ((weight-neutral OR weight-inclusive OR "weight acceptance" OR "size acceptance" OR "body acceptance" OR non-diet OR anti-diet )) AND ((overnutrition* ) OR (ALL=Obesity OR Obes* OR ALL="Obesity, Abdominal" OR "Abdominal obesity" OR ALL="Obesity, Morbid" OR "Morbid Obesity" ) OR (adipos* OR anthropometr* OR "Waist hip ratio" OR "skinfold thickness" )) AND (ALL="Epidemiologic Studies" OR "Epidemiologic Studies" OR "Epidemiologic Study" OR ALL="Cohort Studies" OR "Cohort Studies" OR "Cohort Study" OR "Historical Cohort Studies" OR "Historical Cohort Study" OR "Cohort Analyses" OR "Cohort Analysis" OR "Closed Cohort Studies" OR "Closed Cohort Study" OR "Incidence Studies" OR "Incidence Study" OR ALL="Longitudinal Studies" OR "Longitudinal Studies" OR "Longitudinal Study" OR "Longitudinal Survey" OR "Longitudinal Surveys" OR ALL="Follow-Up Studies" OR "Follow-Up Studies" OR "Follow-Up Study" OR "Followup Studies" OR "Followup Study" OR ALL="Prospective Studies" OR "Prospective Studies" OR "Prospective Study" OR ALL="Case-Control Studies" OR "Case-Control Studies" OR "Case-Control Study" OR "Case-Comparison Studies" OR "Case-Comparison Study" OR "Case-Compeer Studies" OR "Case-Compeer Study" OR "Matched Case-Control Studies" OR "Matched Case-Control Study" OR "Nested Case-Control Studies" OR "Nested Case-Control Study" ) |
| SciELO | (("Health at every size") OR ("Health-at-every-size") OR ("HAES")) AND (("weight-neutral" OR "weight-inclusive" OR "weight acceptance" OR "size acceptance" OR "body acceptance" OR "non-diet" OR "anti-diet")) AND (("overnutrition*") OR ("Obesity" OR "Obes*" OR "Abdominal obesity" OR "Morbid Obesity") OR ("adipos*" OR "anthropometr*" OR "Waist hip ratio" OR "skinfold thickness")) AND ("Epidemiologic Studies" OR "Epidemiologic Study" OR "Cohort Studies" OR "Cohort Study" OR "Historical Cohort Studies" OR "Historical Cohort Study" OR "Cohort Analyses" OR "Cohort Analysis" OR "Closed Cohort Studies" OR "Closed Cohort Study" OR "Incidence Studies" OR "Incidence Study" OR "Longitudinal Studies" OR "Longitudinal Study" OR "Longitudinal Survey" OR "Longitudinal Surveys" OR "Follow-Up Studies" OR "Follow-Up Study" OR "Followup Studies" OR "Followup Study" OR "Prospective Studies" OR "Prospective Study" OR "Case-Control Studies" OR "Case-Control Study" OR "Case-Comparison Studies" OR "Case-Comparison Study" OR "Case-Compeer Studies" OR "Case-Compeer Study" OR "Matched Case-Control Studies" OR "Matched Case-Control Study" OR "Nested Case-Control Studies" OR "Nested Case-Control Study") |
